# Spontaneous Abortion Associated with Zika Virus Infection and Persistent Viremia 

**DOI:** 10.3201/eid2405.171479

**Published:** 2018-05

**Authors:** Anna Goncé, Miguel J. Martínez, Elena Marbán-Castro, Adela Saco, Anna Soler, Maria Isabel Alvarez-Mora, Aida Peiro, Verónica Gonzalo, Gillian Hale, Julu Bhatnagar, Marta López, Sherif Zaki, Jaume Ordi, Azucena Bardají

**Affiliations:** BCNatal–Barcelona Center of Maternal-Fetal and Neonatal Medicine, Barcelona, Spain (A. Goncé, M. López);; ISGlobal, Hospital Clínic–Universitat de Barcelona, Barcelona (M.J. Martínez, E. Marbán-Castro, J. Ordi, A. Bardají);; Hospital Clínic, Barcelona (M.J. Martínez, A. Saco, A. Peiro, V. Gonzalo, J. Ordi);; Hospital Clínic, IDIBAPS and CIBERER, Barcelona (A. Soler, M.I. Alvarez-Mora);; Centers for Disease Control and Prevention, Atlanta, Georgia, USA (G. Hale, J. Bhatnagar, S. Zaki)

**Keywords:** Zika virus, spontaneous abortion, viremia, pregnancy, viruses, Dominican Republic, Spain

## Abstract

We report a case of spontaneous abortion associated with Zika virus infection in a pregnant woman who traveled from Spain to the Dominican Republic and developed a rash. Maternal Zika viremia persisted at least 31 days after onset of symptoms and 21 days after uterine evacuation.

Evidence regarding the association of Zika virus infection and pregnancy loss (spontaneous abortions and stillbirths) has been reported recently (*1*). Zika virus has been detected by reverse transcription PCR (RT-PCR) in brain tissue samples from stillborn infants and from placental tissue obtained from pregnancy losses (*2*,*3*). We report a case of early pregnancy loss associated with Zika virus with evidence of persistent maternal viremia after uterine evacuation.

In mid-June 2016, a 22-year-old woman, who was in the seventh week of gestation, traveled from Spain to the Dominican Republic. Fifteen days after her arrival, she developed a mild macular rash and malaise that resolved after 3 days (Figure). One day after her return to Spain (at 10.5 weeks of pregnancy and 9 days after the onset of symptoms), a routine first-trimester prenatal scan showed an embryo without cardiac activity and a crown–rump length of 19 mm, compatible with a pregnancy loss at an estimated gestational age of 8 weeks and 4 days (Figure). On July 5, 2016, a maternal serum sample tested positive for Zika virus by a commercial real-time RT-PCR with a cycle threshold (C_t_) value of 33, and a urine sample was negative by real time RT-PCR (details on laboratory testing in Technical Appendix). We detected Zika virus IgM and IgG by a commercial immunofluorescence assay (Technical Appendix).

**Figure:**
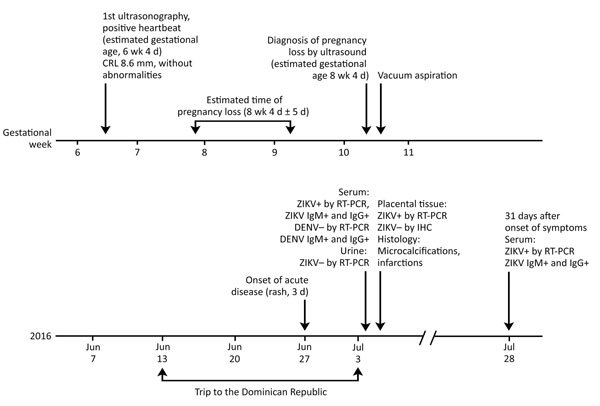
Clinical timeline for a 22-year-old pregnant woman who had suspected Zika virus infection. The woman was in the seventh week of gestation when she traveled from Spain to the Dominican Republic. CRL, crown–rump length; DENV, dengue virus; Ig, immunoglobulin; IHC, immunohistochemistry; ZIKV, Zika virus; RT-PCR, reverse transcription PCR; +, positive; –, negative.

The patient was offered a chorionic villi sampling; the genetic analysis was normal. Surgical evacuation of the uterus was performed by vacuum aspiration followed by curettage. We detected Zika virus by real time RT-PCR in both the transport medium in which the chorionic biopsy was stored (C_t_ = 36) and the supernatant of the karyotype cell culture (C_t_ = 12). Differences in real-time PCR C_t_ values can be explained by active viral replication in the karyotype cell culture. We used the supernatant of the karyotype cell culture to inoculate Vero cells, where we observed a cytopathic effect. We confirmed virus isolation by subsequent infection of new Vero cells, RT-PCR analysis, and sequencing of the Zika virus envelope gene. This analysis suggested active Zika virus replication in embryonic cells. We also detected Zika virus by real time RT-PCR in fresh placental tissue samples from vacuum aspiration (Technical Appendix).

Formalin-fixed paraffin-embedded placental tissues were also analyzed at the Centers for Disease Control and Prevention (CDC; Atlanta, GA, USA). Histopathological analyses of these placental tissues revealed perivillous fibrinoid deposition, focal coarse calcifications, and moderate increase of Hofbauer cells. The histological sections of the placental tissue, which were stained with hematoxylin and eosin, showed a focus of villous necrosis associated with calcifications. A small portion of embryonic membranes was visible, showing no noteworthy inflammatory infiltrate. Immunohistochemical testing on placental tissue did not show presence of Zika virus-specific immunostaining. The histological findings were not relevant to the diagnosis. No specific changes were observed, neither associated inflammation was identified, and only nonspecific mild abnormalities were present. Nevertheless, Zika virus RT-PCR assays and sequencing performed on RNA extracted from placental tissues identified the presence of Zika virus in the sample (*4*). On July 6, 21 days after vacuum aspiration and 31 days after the onset of symptoms, we detected Zika virus in maternal serum samples using RT-PCR (C_t_ = 37).

Our investigation found evidence of Zika virus infection in tissue samples from an early pregnancy loss in a mother infected with Zika virus in the first trimester of pregnancy. Testing of tissues from vacuum aspiration and from chorionic villi sampling revealed that placenta and chorion contained Zika virus RNA. Isolation of Zika virus from the karyotype cell culture confirmed active viral replication in embryonic cells. All the tests performed suggest that the spontaneous abortion in this woman was likely associated with a symptomatic Zika virus infection occurring early in pregnancy. These findings provide further evidence of the association between Zika virus infection early in pregnancy and transplacental infection, as well as embryonic damage, leading to poor pregnancy outcomes (*2*). Given that embryo loss had probably occurred days before maternal-related symptoms, we hypothesize that spontaneous abortion happened early during maternal viremia. The prolonged viremia in the mother beyond the first week after symptom onset concurs with other recent reports (*1*,*5*). However, persistent viremia 3 weeks after pregnancy outcome has not been described previously and underscores the current lack of knowledge regarding the persistence of Zika virus infection. Because we identified Zika virus RNA in placental tissues, our findings reinforce the evidence for early gestational placental tissue as the preferred target for viral tropism (*2*,*4*). Finally, although laboratory tests were performed to dismiss other maternal infections (see online Technical Appendix), the attribution of Zika virus as the cause of the spontaneous abortion must be interpreted with caution, because a non–Zika-related etiology cannot be entirely ruled out. Further studies are warranted to investigate the natural history of Zika virus infection in pregnant women.

Technical Appendix Details of laboratory testing for a 22-year-old pregnant woman with suspected Zika virus infection, 2016.
