# Supplementary material for: Spontaneous Abortion Associated with Zika Virus Infection and Persistent Viremia
Source: Emerg Infect Dis. 2018 May;24(5):933–5. doi: 10.3201/eid2405.171479 (PMC5938776; doi:10.3201/eid2405.171479)
Supplement: Technical Appendix — Details of laboratory testing for a 22-year-old pregnant woman with suspected Zika virus infection, 2016. [file 17-1479-Techapp-s1.pdf]

Article DOI: https://doi.org/10.3201/eid2405.171479

Spontaneous Abortion Associated with Zika Virus Infection and Persistent Viremia after Uterine Evacuation

Technical Appendix

Details of Laboratory Testing

Molecular Diagnostics and Antibody Testing

We tested serum and urine samples for Zika virus using a commercial real-time RT-PCR kit (Real Star Zika RT-PCR kit, Altona Diagnostics, Hamburg, Germany). We detected antibodies against Zika virus using a commercial immunofluorescence test (Arbovirus Fever Mosaic 2, Euroimmun, AG, Lübeck, Germany). We used a commercial assay (Panbio ELISA, Alere, Brisbane, Australia) to test for antibodies against dengue virus (IgG and IgM).

Zika Virus Culture

We inoculated samples onto subconfluent Vero cells in 25 cm^2 culture flasks and maintained them in Dulbecco’s modified Eagle medium (DMEM) supplemented with 2.5% fetal bovine serum (FBS). We inspected the cells visually every day for the presence of cytopathic effect (CPE). The culture medium of the 25 cm^2 flask presenting CPE was centrifuged at 1000 rpm for 5 minutes to pellet detached cells and we used the supernatant to inoculate new Vero cells. The supernatant of this cell culture presented a similar CPE and tested positive for Zika virus by real-time RT-PCR.

Immunohistochemical Testing

Immunohistochemical analysis was performed at the US Centers for Disease Control and Prevention. A mouse polyclonal anti-Zika virus antibody and a polymer-based indirect colorimetric immunoalkaline phosphatase detection system with fast red chromogen (Thermo Fisher Scientific, Runcorn, Cheshire, UK) were used. Deparaffinized and rehydrated tissue sections were placed in a LAB Vision autostainer and digested in 0.1 mg/mL proteinase K (Roche Diagnostics, Mannheim, Germany). Tissue sections were incubated with anti-Zika

antibody for 30 minutes, followed by sequential incubations with MACH 4 Universal AP Polymer Kit (Biocare Medical LLC, Concord, CA, USA) and fast red substrate (Dako North America, Carpinteria, CA, USA). Sections were then counterstained in Mayer's modified hematoxylin (Poly Scientific R&D Corp. Bay Shore, NY, USA) using the Sakura Automatic Slide Stainer and mounted with aqueous mounting medium (Polysciences, Warrington, PA, USA) (*1*).

Placental Pathology

Pathological evaluation was performed after formalin-fixed paraffin-embedded sections of placental tissue were processed. Histopathological analyses of placental samples were performed at CDC and the Pathology Department of the Hospital Clinic of Barcelona. Histopathological analyses of the placental tissues showed normal findings consistent with intrauterine embryonic death. They revealed perivillous fibrinoid deposition, focal coarse calcifications, and moderate increase of Hofbauer cells. A focus of villous necrosis associated with calcifications was also noted. A small portion of embryonic membranes was seen showing no notable inflammatory infiltrate.

Genetics: Microarray Analysis

The patient was offered and received a chorionic villi sampling for evaluation of chromosomal and submicroscopic anomalies. The sample was cleaned under the dissecting microscope and maternal decidua was rejected. Direct chromosome analysis showed a normal 46,XX karyotype, and molecular analysis by quantitative fluorescence PCR did not show the presence of a second cell line of maternal origin. The array-comparative genomic hybridization (aCGH) (qChipCM 8x60K, qGenomics, Barcelona, Spain) revealed a normal genetic analysis with a feminine profile, arr (1–22,X)x2.

We isolated DNA from embryonic tissue automatically with the MagNA Pure Compact Instrument (Roche, Indianapolis, IN, USA) using the MagNA Pure Nucleic Acid isolation kit according to the manufacturer's instructions. We measured DNA concentration with a Qubit fluorometer (ThermoFisher Scientific, Wilmington, DE, USA).

We performed whole-genome array CGH using the qChipCM microarray following the manufacturer's instructions (ChipCM 8x60K, qGenomics, Spain). This microarray has a 350–500 kb resolution along the whole genome, a resolution of 100–125 kb in subtelomeric and

pericentromeric regions, and ≈30 kb resolution in constitutional regions associated with pathology. This microarray applies University of California Santa Cruz Genome Browser hg18 (NCBI Build 36, Mar 2006; http://www.qgenomics.com/es/servicios/asistenciales/servicios-con-microarrays/postnatal-qchip-postr) as the reference assembly. Briefly, we labeled, purified, precipitated, and hybridized 250 ng of patient DNA and of a sex-matched control according to manufacturer's instructions (qGenomics). We analyzed this array using a scanner (Agilent G2565CA Microarray Scanner System, Agilent Technologies, Santa Clara, CA, USA). We quantified and analyzed the captured images using Cytogenomics software (Agilent Technologies).

Diagnostics to Rule Out Other Maternal Infections

According to protocols for prenatal care, and as routinely performed for all pregnant women in the first trimester, the patient was screened for rubella and toxoplasma (she showed previous immunity to both infections), syphilis, HIV, and hepatitis B (she showed negative results). To rule out other maternal infections, residues from curettage were tested and showed negative results by RT-PCR for herpes simplex virus (HSV) type 1 and type 2. Endocervix samples were collected 31 days after surgical evacuation and tested negative for Zika virus by RT-PCR assay, as well as for *Chlamydia trachomatis*, *Neisseria gonorrhoeae*, and HSV 1 and 2.

Reference

1. Martines RB, Bhatnagar J, de Oliveira Ramos AM, Davi HP, Iglezias SD, Kanamura CT, et al. Pathology of congenital Zika syndrome in Brazil: a case series. Lancet. 2016;388:898–904. http://dx.doi.org/10.1016/S0140-6736(16)30883-2
